# A Multifunctional MXene/PVA Hydrogel as a Continuous Ionic Thermoelectric Generator and a Strain/Temperature Sensor

**DOI:** 10.1002/smll.202407529

**Published:** 2024-11-20

**Authors:** Dezhuang Ji, Baosong Li, Dawei Zhang, Balamurugan Thirumal Raj, Moh'd Rezeq, Wesley Cantwell, Lianxi Zheng

**Affiliations:** ^1^ Department of Mechanical and Nuclear Engineering Khalifa University of Science and Technology P.O. Box Abu Dhabi 127788 UAE; ^2^ Department of Aerospace Engineering Khalifa University of Science and Technology P.O. Box Abu Dhabi 127788 UAE; ^3^ Research & Innovation Center for Graphene and 2D Materials (RIC‐2D) Khalifa University of Science and Technology P.O. Box Abu Dhabi 127788 UAE; ^4^ Department of Physics Khalifa University of Science and Technology P.O. Box Abu Dhabi 127788 UAE; ^5^ System on Chip Center Khalifa University of Science and Technology P.O. Box Abu Dhabi 127788 UAE; ^6^ Research and Innovation on CO_2_ and H_2_ Center (RICH) Khalifa University of Science and Technology P.O. Box Abu Dhabi 127788 UAE

**Keywords:** energy harvesting, hydrogel, Ionic thermoelectric, MXene, sensor

## Abstract

This research reports a continuous output ionic thermoelectric (*i*‐TE) system based on MXene/PVA (polyvinyl alcohol) hydrogel, by utilizing thermo‐diffusion of Cu^2+^ and Cl^−^ ions and the redox reaction involving Cu/Cu^2+^ at the electrode interfaces. The thermopower of the *i*‐TE system can be independently tuned to a value of −3.13 mVK^−1^ by adjusting the ion diffusivity via MXene (Ti_3_C_2_T*_x_*). The *i*‐TE system demonstrates a rapid response time of less than 100 s, outperforming any other polyelectrolyte‐based system. Crucially, the *i*‐TE system achieves continuous current output when equipped with copper electrodes, facilitated by the redox reaction involving Cu/Cu^2+^, and maintains stable long‐term outputs across a range of resistances from 1 kΩ to 1 MΩ. A three‐serial‐connected *i*‐TE module demonstrates an output voltage of 26 mV with 6 °C temperature difference, confirming the feasibility of creating an array of *i*‐TE devices for substantial energy output. Beyond energy harvesting, the MXene/PVA hydrogel serves as multifunctional strain/temperature sensors, capable of detecting mechanical strains via the piezoresistive effect and locating finger contact points via the ionic thermoelectric effect.

## Graphical abstract

A continuous‐output ion thermoelectric generator based on the redox reaction of Cu/Cu^2+^ embedded in a flexible MXene/PVA hydrogel is developed to harvest near‐room‐temperature heat. The thermopower is enhanced by reducing the diffusivity of ions via the addition of MXene. The device also functions as a multifunctional strain and temperature sensor.

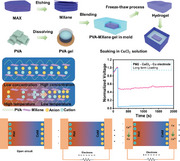

## 1 Introduction

In the modern era, energy is of critical importance, with a particular emphasis on sustainable and clean sources. The thermoelectric (TE) effect, a solid‐state phenomenon that converts thermal energy into electrical energy, offers a viable solution for harnessing low‐grade heat, with advancements in nanotechnology injecting new dimensions into this field. Notably, the group IV‐VI compound SnSe^[^ ^1^, ^2^, ^3^ ^]^ has emerged as a key material, exhibiting promising TE performance. Various innovative strategies, such as the utilization of 3D charge and 2D phonon approaches,^[^ ^4^ ^]^ band manipulation,^[^ ^5^ ^]^ multiband alignment,^[^ ^6^ ^]^ phonon‐electron decoupling,^[^ ^7^ ^]^ and lattice plainification,^[^ ^8^ ^]^ have been employed to enhance its energy harvesting efficiency. Additionally, the extensively studied high‐performance TE material PbTe^[^ ^9^, ^10^, ^11^, ^12^ ^]^ serves as a benchmark for exploring quantum effects associated with TE behavior. Despite their efficacy, both SnSe and PbTe face limitations in terms of low electrical conductivity at near‐room temperatures, primarily attributable to their relatively large bandgaps. This characteristic impedes their application in capturing heat energy at lower temperatures, at which a significant portion of wasted heat is typically found. While narrow band semiconducting materials such as Ag_2_Se^[^ ^13^, ^14^, ^15^, ^16^, ^17^ ^]^ and Bi_2_Te_3_^[^ ^18^, ^19^, ^20^, ^21^ ^]^ have demonstrated a promising TE performance at low temperatures, their Seebeck coefficients still fall short of those ideal levels required for efficient conversion of thermal energy to electricity.

Compared to these electronic thermoelectric (*e*‐TE) systems, ionic thermoelectric (*i*‐TE) systems stand out for their orders of magnitude higher Seebeck coefficients. The TE effect in ionic systems is rooted in the Soret effect, where ions migrate from the hot side to the cold side driven by thermophoresis. However, an increase in ion concentration on the cold side prompts ions to move back toward the hot side, due to concentration gradient (diffusion). The influence of electric fields also interacts with the thermophoresis effect, since ions carry electric charges. Eventually, an equilibrium state is established through the interplay of these three thermodynamic forces, and the Seebeck coefficient is computed based on ‐∆*V*/∆*T*, where ∆*V* represents the voltage difference and ∆*T* denotes the temperature difference. Studies have revealed that the incorporation of NaOH in polyethylene oxide (PEO)^[^ ^22^ ^]^ yields a remarkable Seebeck coefficient of up to 11.1 mVK^−1^, which can be further enhanced to 24 mVK^−1^ with the inclusion of a cellulose matrix.^[^ ^23^ ^]^ Notably, a PVA hydrogel system, enriched with NaOH, exhibits N‐type *i*‐TE behavior, resulting in an impressive Seebeck coefficient of −37.61 mVK^−1^,^[^ ^24^ ^]^ while an infiltration of HCl results in a positive Seebeck coefficient of 38.2 mVK^−1^.^[^ ^25^ ^]^ Furthermore, research indicates that graphene oxide (GO),^[^ ^26^, ^27^ ^]^ with a negatively charged surface, can modulate ion diffusivity, thereby improving the Seebeck coefficient in *i*‐TE systems. Despite these advancements, a key challenge faced by *i*‐TE systems is their inability to achieve continuous current output and they can only be operated as capacitors, because the ions cannot transport across the electrode interfaces to establish a conduction loop.

In addition to periodically switching the heat source or external electrodes for generating a cyclic power,^[^ ^28^ ^]^ thermogalvanic cells make one promising step toward continuous output by employing redox pairs that consume accumulated electrons/holes and transport ions within an *i*‐TE system. The redox pairs [Fe(CN)_6_]^4−^/[Fe(CN)_6_]^3−^ are commonly used as entropy carriers.^[^ ^29^, ^30^, ^31^, ^32^, ^33^ ^]^ However, this continuous current output is realized at the expense of reducing the entropy gradient, because it is difficult to independently tune the entropy of [Fe(CN)_6_]^4−^ and [Fe(CN)_6_]^3−^, given the facts that they are uniformly dissolved in the solvent and must be transported in the opposite directions. In order to increase the entropy difference between [Fe(CN)_6_]^4−^ and [Fe(CN)_6_]^3−^, many additive salts are studied, to enhance the Seebeck coefficient. Han et al. partially addressed this issue by combining the thermophoresis effect and the thermogalvanic effect to achieve a high‐performance *i*‐TE system.^[^ ^34^ ^]^ Nevertheless, further enhancement of the TE performance of [Fe(CN)_6_]^4−^/[Fe(CN)_6_]^3−^ remains challenging, and studying other *i*‐TE systems for continuous operation is crucial for low‐temperature heat harvesting.

In this study, we introduce a novel approach to address this limitation through the development of a flexible and continuous output *i*‐TE system based on a MXene/PVA hydrogel infused with CuCl_2_ salt. The Cu^2+^ and Cl^−^ ions within the system serve as the primary entropy carriers, establishing a voltage difference when subjected to a temperature gradient owing to the thermophoresis effect. Continuous energy output is then achieved via an electrolysis effect by integrating the system with copper electrodes, which consume the accumulated electrons/holes via corrosion. Our experiments further reveal that the oxygen‐containing functional groups in MXene could affect ion diffusivity and thus enhance the thermopower (It is used to replaced Seebeck coefficient since a synergistic effect is observed in our research and this term is more appropriate). The resultant *i*‐TE system showcases its versatility by not only serving as an energy source but also as a multifunctional sensor capable of detecting strains through the piezoresistive effect and pinpointing contact locations or measuring temperatures based on the ionic thermoelectric effect.

## 2 Results and Discussion

### 2.1 Hydrogel Preparation and Characterization

**Figure 1:**
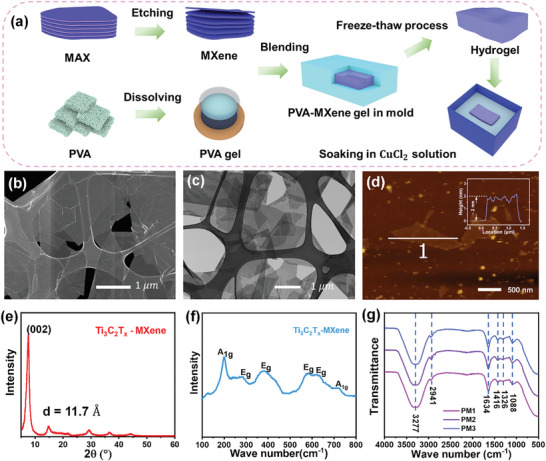
Preparation of MXene/PVA hydrogel and material characterizations. a) Schematic illustration of the preparation process of MXene/PVA hydrogel. b) SEM and c) TEM image of MXene flakes. d) AFM image and thickness distribution of MXene flakes. e) XRD and f) Raman shift spectrum of MXene film. g) FTIR transmission spectrum.

The MXene/PVA hydrogel was prepared using a simple freeze‐thaw method, as schematically shown in **Figure** **1**a. Briefly, MXene dispersion (Figure S1, Supporting Information) was obtained by selectively etching the Al layer in the MAX phase using in‐situ formed HF acid,^[^ ^35^ ^]^ and PVA gel was prepared by dissolving PVA powder in water at 90 °C. Various amounts of MXene dispersions were then mixed with PVA gel and poured into a mold after being degassed. The mold was frozen for 12 h at ‐24 °C and then thawed for 3 h at room temperature. Finally, obtained MXene/PVA hydrogel was soaked in 0.01 M CuCl_2_ solution for 1 h to load thermal energy carriers (ions). The morphology of the MXene sheets was studied using scanning electron microscopy (SEM) and transmission electron microscopy (TEM) (Figure 1b,c). Atomic force microscopy (AFM) images and the corresponding thickness profile of the MXene sheets are shown in Figure 1d. These images confirm that well‐delaminated 2D MXene sheets are obtained, with a size distribution from ≈1 to 4 µm and a thickness of ≈2 nm. Furthermore, MXene film was fabricated using the vacuum filtration method. The X‐ray diffraction (XRD) pattern and Raman shift spectrum were investigated to study the properties of the synthesized MXene. A sharp (002) peak is observed in the XRD pattern (Figure 1e), featured by successive peaks of (004) and (008), highlighting the 2D geometry of MXene sheets. The employed X‐ray wavelength is 1.54184 Å, leading to a lattice constant of 11.7 Å in the 2D MXene unit cell. Additionally, MXene exhibits two Raman‐sensitive modes: E_g_, an in‐plane vibration of Ti and C atoms, and A_1g_, an out‐of‐plane vibration of Ti and C atoms.^[^ ^36^ ^]^ Surface functional groups were reported to distort these vibrational modes and the Raman absorption spectrum. Two distinct A_1g_ peaks are observed at 202 and 720 cm^−1^, corresponding to the out‐of‐plane vibration of the Ti, O, and C atoms, respectively (Figure 1f). The suppression of the 720 cm^−1^ peak is likely due to the stacking of MXene sheets, which restricts the out‐of‐plane mode. The in‐plane vibration mode between 230–470 cm^−1^ is induced by surface groups attached to the Ti atoms, and the peaks at 580 cm^−1^ and 620 cm^−1^ are assigned to the in‐plane vibration mode of carbon.

The prepared MXene/PVA hydrogel was characterized using Fourier transform infrared spectroscopy (FTIR) to investigate the presence of functional groups (Figure 1g). Two peaks at high wavenumbers (3277 and 2914 cm^−1^) represent the O─H stretching and C─H stretching modes, respectively.^[^ ^37^ ^]^ The peak at 1634 cm^−1^ represents C═O stretching, while the other peaks correspond to stretching between C and O: OH─C─OH (1416), C─O─C (1326), and C─O─C─OH (1088). The element mapping of the MXene/PVA hydrogel is shown in Figure S2 (Supporting Information). The elements C, O, and Ti originate from the PVA backbone and MXene sheets, while the presence of F is due to the etching of MAX using in‐situ HF acid. The Cu and Cl are from the infiltration of the CuCl_2_ solution; Excess Cl is observed due to the use of HCl acid during the etching of MAX. In addition to the above structural and morphological characteristics, basic mechanical tests indicate that the MXene/PVA hydrogel is highly adhesive and stretchable, capable of withstanding a strain in excess of 200% (Figure S3, Supporting Information).

### 2.2 Mechanical Properties

**Figure 2:**
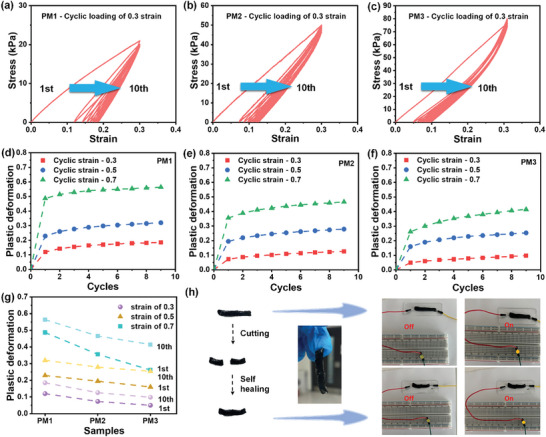
Mechanical properties of the MXene/PVA hydrogels. Cyclic stretching of a) PM1, b) PM2, and c) PM3 hydrogel with a constant strain of 0.3. The permanent strains in (d) PM1, (e) PM2, and (f) PM3 with respect to stretching cycles. g) A comparison of permanent strains in different samples (representing different MXene contents). h) Self‐healing property of the MXene/PVA hydrogel.

Three MXene/PVA hydrogels with different MXene contents were prepared and named as PM1, PM2, and PM3, from low to high MXene content (details can be found in the Experimental Section). Cyclic stretching and compression were performed to investigate their mechanical properties. **Figure** **2**a–c shows the cyclic stretching of MXene/PVA hydrogels at a constant strain of 0.3. For the sample PM1, the stress reaches 21 kPa during the first cycle (Figure 2a). However, the strain does not return to zero when the strain is fully released, leaving a large permanent strain or plastic deformation (≈0.12), and this plastic deformation continuously increases in subsequent stretching cycles. Notably, Young's modulus increases with cyclic stretching, as indicated by a sharper stress‐strain curve. The same phenomena are observed in samples PM2 and PM3, but with higher stresses at the same strain (49 kPa for PM2 and 80 kPa for PM3 at a strain of 0.3). This indicates a higher Young's modulus, showing an increasing trend with higher MXene content. To further investigate these permanent strains, three samples were stretched at strains of 0.5 and 0.7 (Figure S4, Supporting Information), and the relationship between permanent strain and stretching cycles was investigated (Figure 2d–f). A sharp increase in permanent strains is observed in PM1 (Figure 2d) after the first cycle for all applied strains (0.12, 0.23, and 0.49 for strains of 0.3, 0.5, and 0.7, respectively). The permanent strains continue increasing until reaching an almost constant value, with the rate of increase significantly reduced. This trend also applies to PM2 and PM3 (Figure 2e), but with lower permanent strains. The permanent strains after the first cycle are 0.073, 0.195, and 0.356 for PM2, and are 0.05, 0.16, and 0.262 for PM3, at strains of 0.3, 0.5, and 0.7 respectively. A decreasing trend in permanent strain is observed in passing from PM1 to PM3 (Figure 2d–f), indicating that a higher MXene content mitigates permanent strains (or plastic deformation) in the MXene/PVA hydrogels. It can be concluded from Figure 2g that high strains and large strain cycles both lead to high levels of plastic deformation, but stretching strains dominate plastic deformation because they primarily occur in the first cycle.

From the mechanical tests, two key conclusions were drawn. First, increasing the MXene content in the PVA hydrogel significantly enhances its Young's modulus, indicating greater stiffness and resistance to elastic deformation under stress. Second, as MXene content increases, plastic deformation is reduced during cyclic loading. The observed increase in Young's modulus with repeated stretching and plastic deformation of PVA hydrogel can be attributed to its intrinsic structure and behavior. When a hydrogel is subjected to stress, the polymer chains tend to slide in the direction of the applied force. With repeated stretching, this process promotes alignment of the polymer chains along the stretch axis, which strengthens the material but also causes irreversible changes in PVA chain movement, leading to plastic deformation. The addition of MXene to the PVA matrix provides structural reinforcement. MXene sheets, as 2D nanomaterials with excellent mechanical properties, act as strong and rigid components that bridge the gaps between the PVA polymer chains. This creates a network of connections between polymer chains, restricting their movement when external forces are applied. As a result, the Young's modulus is enhanced. Incorporating MXene into the hydrogel matrix strengthens the overall hydrogel through this reinforcement strategy. The increased Young's modulus directly correlates with MXene content, indicating a clear relationship between MXene concentration and the hydrogel's mechanical properties. Despite the increased stiffness, the MXene sheets introduce a new mechanical interaction that limits the alignment of PVA chains during stretching. The reduced chain mobility is due to the increased interconnection and restriction of PVA chains by the MXene sheets. Thus, MXene sheets play a crucial role in reducing plastic deformation. By acting as mechanical barriers, they resist the sliding motion of PVA chains during cyclic stretching, which reduces irreversible alignment of PVA chains leading to a reduced plastic deformation.

In addition to the stretching tests, cyclic compression was conducted on the samples (Figure S5, Supporting Information). Both plastic deformation and reinforcement effects from MXene were observed. The MXene/PVA hydrogel is also highly adhesive and exhibits self‐healing properties. When a strip of MXene/PVA hydrogel is cut into two parts, it can self‐heal into a whole strip within a few minutes if the parts are pressed together (Figure 2h). The self‐healed sample is nearly identical to the original and can be used to connect a circuit and light up a LED bulb.

### 2.3 Thermoelectric Properties

**Figure 3:**
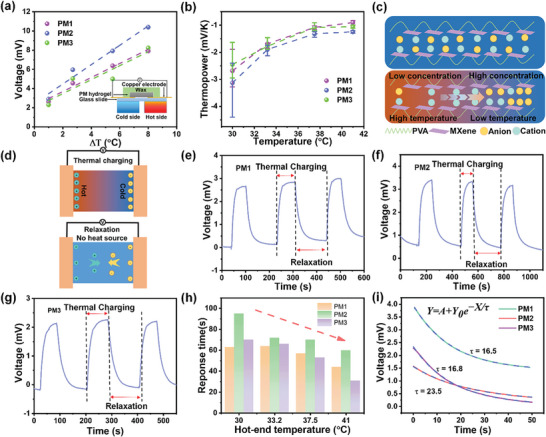
Thermoelectric behavior and transient analysis of MXene/PVA hydrogels. a) Temperature gradient generated TE voltage with respect to temperature difference and an inset schematically showing the experimental setup for testing the thermopower. b) Average thermopower with respect to temperature of the MXene/PVA hydrogels. c) Illustration of the mechanism of ionic thermoelectric effect. d) Schematic illustration of thermal charging/relaxation. The voltage variation with respect to time in cyclic thermal charging/relaxation for samples e) PM1, f) PM2, and g) PM3. h) Response time during thermal charging for PM1, PM2, and PM3 from 30 – 41 °C. i) Voltage variation with respect to time during thermal relaxation, and the respective exponential‐fitting curves.

The thermoelectric performance of the MXene/PVA hydrogels was investigated using the experimental set‐up schematically shown in the inset of **Figure** **3**a. A MXene/PVA hydrogel was attached between two copper electrodes on a glass slide and sealed with wax to prevent water evaporation during testing. To create a temperature difference, one side was attached to a hot plate with a controllable temperature, while the other side was suspended in air to serve as a cold source. A thermocouple was used to precisely measure the temperatures on both sides, as the temperature of the cold side was not at room temperature due to heat transfer. The voltage generated by the ionic thermoelectric effect with respect to the temperature gradient was measured and the results are shown in Figure 3a. It is observed that the TE voltage increases almost linearly with the temperature difference. The sample PM2 exhibits the highest TE voltage, increasing from 3 to 10.5 mV as the temperature difference changes from 1 to 8 °C. The performance of PM1 and PM3 samples was similar, with the TE voltage increasing from ≈2.5 to 7.6 mV in the same temperature range. To verify the reliability of our experiments, more independent experiments were conducted for PM1, PM2, and PM3, respectively (Figure S6, Supporting Information). The tests were carried out under identical conditions. Thermoelectric voltage was recorded over time, and temperature differences were measured using a two‐channel thermocouple. From the results shown in the figure, one can see reasonably stable thermal voltages, with relatively larger variations at low temperature gradients but much smaller variations at larger temperature gradients.

The average thermopowers, *S*, were then calculated from ‐∆*V*/∆*T*, and are shown in Figure 3b. The thermopower is plotted against the temperature at the hot side to emphasize their near‐room‐temperature potential. PM2 still offers the best performance, with an average thermopower of −3.13 mVK^−1^ at a hot side temperature of 30 °C, compared to −2.67 and −2.44 mVK^−1^ for PM1 and PM3, respectively. A downward trend in *S* is observed with increasing temperature. The thermopower decreased to −1.23 mVK^−1^ at 41 °C for PM2, and to ≈−1 mVK^−1^ for PM1 and −0.9 mVK^−1^ PM3.

Figure 3c schematically illustrates the mechanism of the ionic thermoelectric effect in the MXene/PVA hydrogel system. Both anions and cations are initially random and uniform in hydrogel. However, when a temperature gradient is applied, both types of ions move from the hot side to the cold side due to the thermophoresis effect, leading to an increased ion concentration on the cold side. Dynamically, the induced concentration gradient, as well as the induced electric field, will cause ions to move back from the cold side to the hot side. An equilibrium state is reached when the thermophoresis force balances the forces from the concentration gradient and electrical forces. In our system, there are two main charged ions Cu^2+^ and Cl^−^, which result in a positive thermopower (*S^+^*) and a negative thermopower (*S^−^*), with the net thermopower being *S^+^* + *S^−^*. Since Cl^−^ has a lower hydration energy than Cu^2+^ and is thus more thermally sensitive, the absolute value of *S^−^* is higher than *S^+^*, resulting in a negative net thermopower (N‐type *i*‐TE system). Consequently, our MXene/PVA hydrogel containing CuCl_2_ salt is an N‐type *i*‐TE system, primarily resulting from the difference in thermal gradient migration between cations and anions.

The presence of MXene could tune the diffusivity of ions through interactions to enhance the thermopower. Cyclic thermal charging/relaxation (contacting/removing the heat source) experiments were conducted (Figure 3d) to investigate the relationship between the ionic thermoelectric effect and the ion diffusivity. As shown in Figure 3e–g, the TE voltage increases over time when the heat source is attached, until reaching a stable state. Once the heat source is removed, the voltage difference across the electrodes begins to decrease due to a Fick diffusion process driven by the concentration gradient. The response times during thermal charging of the three MXene/PVA hydrogels with respect to temperature are compared in Figure 3h. PM2 shows the longest response time of 95 s at 30 °C, while PM1 and PM3 exhibit faster responses, with values of 63 and 70 s, respectively. As the temperature increases, all samples respond faster, requiring a shorter time to reach a stable state. At 41 °C, only 60 s are required for PM2, compared to 44 s for PM1 and 31 s for PM3. The faster response time versus temperature is attributed to the higher ion diffusivity at elevated temperatures. At any given temperature, PM2 always exhibits a significantly longer response time compared to PM1 and PM3 within tested temperature ranges. This suggests that ions in PM2 have much lower diffusivity, which must arise from the interaction between the ions and the MXene surfaces. To further verify the diffusion phenomenon and minimize the influence of the temperature gradient, the relaxation/equilibrium stage was investigated. According to Fick diffusion theory, the voltage variation with respect to time can be approximately resolved analytically if the initial conditions are known. Using an exponential equation *Y* = *A* + *Y_0_e^−X/τ^* to fit the relaxation/equilibrium process, it is determined that the *τ* factor represents the relaxation time and is directly related to the ion diffusivity (details can be found in the **Methods**). The relaxation parameter at 30 °C is extracted (Figure 3i), revealing that PM2 has an *τ* factor of 23.5 s, while PM1 and PM3 have lower *τ* factors of 16.5 and 16.8 s, respectively. To verify statistical credibility, more results of relaxation time obtained from 30 to 41 °C (Figure S7, Supporting Information) are summarized in Table S1 (Supporting Information) and their averages are compared in Figure S8 (Supporting Information), which shows strong consistency in that the PM2 possesses the highest average *τ*. Despite heating at various temperatures, the *τ* factors show a similar value for the same sample, suggesting relaxation process is governed by the Fick's diffusion under concentration gradient. Overall, the cyclic thermal charging/relaxation results are consistent with the proposed mechanism.

To investigate the conductivity of the hydrogels, electrochemical impedance spectroscopy (EIS) measurements were conducted with a two‐electrode system. As shown in Figure S9 (Supporting Information) (lower part), single semicircles are observed, suggesting a simple porous structure with uniform ion transport pathways, which in contrast to systems with complex microstructures that would exhibit multiple semicircles due to varied diffusion resistances from multiple phase boundaries.^[^ ^38^, ^39^ ^]^ Furthermore, the absence of a 45° Warburg impedance at low frequencies indicates that the system does not follow the classical model of semi‐infinite linear diffusion, where ions diffuse into an infinite volume. Instead, the system operates under a finite diffusion regime, characterized by two distinct boundaries that define steady‐state diffusion.

To further analyze the EIS data, an equivalent circuit is proposed and shown in Figure S9 (Supporting Information) (upper part). A parallel connected resistor R2 and constant phase element (CPE) is employed to simulate electrode‐electrolyte interface. R2 is controlled by two process: mass transport and charge transfer, which refers to reaction species transportation inside the diffusion layer as well as the electrons transfer between redox species and electrode surface.^[^ ^40^ ^]^ The CPE is employed to simulate the non‐ideal capacitance result from surface roughness. Additionally, a series resistor R1 is used to account for total resistance from sources such as the electrolyte, the MXene/PVA composite matrix, and contact resistance.

According to this equivalent circuit, R2 could be obtained from the radius of the semicircle, which is the resistance from mass transport and charge transfer process.^[^ ^41^, ^42^ ^]^ It is observed that PM2 has the largest R2. Since the charge transfer resistance is governed by the electrode surface reaction kinetics, that is, reaction rates, which shows minimal difference in Cu/Cu^2+^ systems, the increase in R2 is primarily attributed to a rise in mass transport resistance. This is due to reduced diffusivity, which slows the mass transfer process in the diffusion layer. This observation is consistent with the mass diffusion‐controlled process from CV analysis at low scan rates, where reduced diffusivity leads to a significant increase in equivalent resistance. Therefore, it is concluded that lower diffusivity will lead to an increase of R2. The corresponding conductivities are then calculated based on samples’ size and geometry, summarized in **Table** **1**. PM2 shows the lowest conductivity, which is consistent with our earlier statement that MXene reduces the ionic diffusivity.

**Table 1:** Conductivity of PM1, PM2 and PM3.

Sample	Conductivity [mSm^−1^]
PM1	18.8
PM2	12.7
PM3	16.2

The impact of ion/MXene interaction and the diffusion‐controlled mechanism could be explained in a more detailed way. The thermoelectric effect in ionic systems is induced by the Soret effect, where ions migrate under a temperature gradient (so termed as temperature‐driven migration), leading to ion accumulation. At the same time, the ion accumulation creates a concentration gradient, causing ions to migrate in the opposite direction (termed as concentration‐induced diffusion).^[^ ^43^ ^]^ At equilibrium, the net ion flux is zero, expressed as: 0 = *D*ΔC – *D_T_* Δ*T*. Here, *D* represents the ion diffusivity during concentration‐induced diffusion process, while *D_T_* denotes thermophoresis mobility during temperature‐driven migration. This equation can be rewritten as ΔC/Δ*T* = *D_T_*/*D*. In a thermoelectric system, ions are charged, and high thermopower implies a significant ion accumulation on one side, resulting in a large voltage difference. Thus, the concentration gradient is directly proportional to the thermopower, which evaluates the equilibrium of the system. According to this equation, the thermopower is proportional to the temperature‐driven mobility (*D_T_*) and inversely proportional to the concentration‐induced diffusivity (*D*).

For a thermoelectric system with a selected redox pair, tuning temperature‐driven migration (thermophoresis mobility) becomes impossible, because it relates to change the ion type or ion entropy. The easiest way in improving thermoelectric performance is to tune the concentration‐induced diffusivity. Therefore, in this study, MXene is added in hydrogel to reduce the concentration‐induced diffusivity. MXene surfaces have a lot of functional groups that carry negative charges, evidenced by a zeta potential of ≈−35.4 mV in water.^[^ ^44^ ^]^ These charges influence the motion of ions in solution through electrostatic interactions, largely reducing their concentration‐induced diffusivity and thus improving the thermopower of the system.

Above discussed relaxation process (Figure S8; Table S1, Supporting Information) could be considered as a direct evaluation of the concentration‐induced diffusivity. In such a relaxation process the temperature gradient is removed, so that the decay of the thermal voltage solely depends on the concentration‐induced diffusion. PM2 exhibits the longest relaxation time, indicating the slowest relaxation and, therefore, the lowest diffusivity. The exact physical model of ion diffusivity in hydrogels remains incomplete and is an active area of research. In literature, ion diffusivity in hydrogel is found to be influenced by both ion‐matrix interactions and water content.^[^ ^45^ ^]^ At low MXene content, increasing MXene concentration significantly reduces ion diffusivity via electrostatic interactions, as observed in the transition from PM1 to PM2. However, at higher MXene content, the water content increases, as MXene enhances the PVA hydrogel's water retention ability due to its interconnected network, leading to improved ion diffusivity.^[^ ^46^, ^47^ ^]^ This explains why PM3 shows a higher ion diffusivity and correspondingly lower thermopower compared to PM2.

### 2.4 Continuous Output of MXene/PVA Hydrogel‐Based *i*‐TE Devices

**Figure 4:**
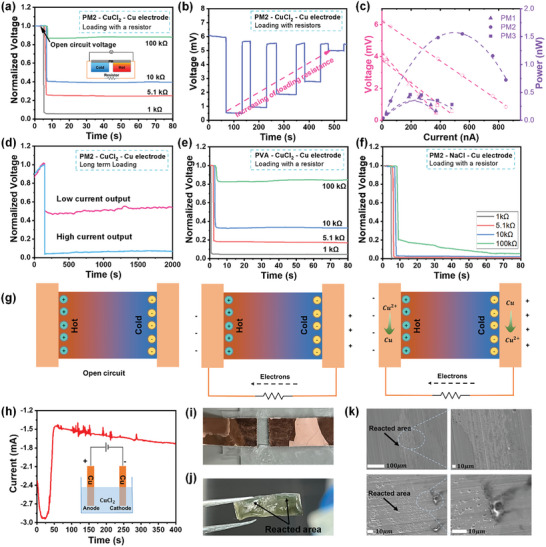
Continuous current output of MXene/PVA hydrogel‐based *i*‐TE device. a) Normalized voltage of PM2 under different external loading (1, 5.1, 10, and 100 kΩ). b)The output voltage with respect to time with stepwise changed resistance (1, 2, 5.1, 10, and 100 kΩ in sequence). c) The output voltage and power analysis of PM1, PM2, and PM3 under (Δ*T* = 2 K). d) Long‐term stability test of PM2 *i*‐TE device under low current discharging (with 10 kΩ load) and high current discharging (with 1 kΩ load). The voltage variation of e) *i*‐TE device with pure PVA hydrogel infiltrated CuCl_2_ solution with Cu electrodes and f) *i*‐TE device with PM2 hydrogel infiltrated NaCl solution with Cu electrodes when the external resistance of 1, 5.1, 10, and 100 kΩ are connected. g) Principle illustration of continuous current output. h) Current with respect to time under 2 mV bias for the electrolytic cell consists of Cu electrodes and 0.01 M CuCl_2_ electrolyte. The optical image of i) reacted electrode and j) reacted MXene/PVA hydrogel after continuous operation. k) SEM images of reacted electrodes.

The prepared MXene/PVA hydrogel demonstrates continuous current output when integrated with Cu electrodes. As shown in **Figure** **4**a, in which the normalized voltage (real voltage divided by open circuit voltage) is used, an immediate voltage drop is observed when an external load is connected, resembling an “internal resistance”. This voltage reduction increases with the decrease of external resistance. A 100 kΩ resistance results in almost a 12% reduction in normalized voltage, while a reduction of more than 90% in voltage is observed when a 1 kΩ load is connected. It is easy to estimate that the internal resistance is ≈10 kΩ, since nearly a 50% voltage reduction is observed at this loading resistance. Despite the voltage drop, a stable and continuous current output is observed at a reduced voltage, which is very different from other capacitive *i*‐TE devices whose voltage decreases to 0 over time when an external circuit is connected (due to the fact that no current flow within the *i*‐TE devices and electrons move from the negative to positive side will screen the electric field generated by ions). The continuous output performance was further studied by changing the load resistance from 1 to 100 kΩ in a stepwise process (Figure 4b). It is observed that the voltage drops immediately when a resistor is connected, but returns instantly to its open circuit value upon disconnection. As the external resistance decreases, the voltage drops more, but it always returns to its original value upon disconnection. The output power and voltage under a 2K temperature difference were measured and analyzed. As shown in Figure 4c, PM2 exhibits the highest open circuit voltage (6.2 mV) and the maximum output power of 1.54 nW at 3.05 mV. Finally, to verify the long‐term output stability of our *i*‐TE device, two discharging tests are conducted at relatively low and high discharge currents for 2000 s (Figure 4d), and no further voltage reduction was observed during the discharging period in both cases, confirming its continuous and stable operation.

Additional experiments were conducted to reveal the principle behind the continuous operation. First, a pure PVA hydrogel was prepared to eliminate the influence of MXene. The *i*‐TE device with pure PVA hydrogel was tested under the same loading conditions. As shown in Figure 4e, it behaves similarly, and the continuous output is observed under all loading resistances, suggesting that MXene is not the cause for the continuous operation although it could boost the thermoelectric coefficient. Second, we checked the electrolyte with sodium chloride (NaCl) replacing CuCl_2_, and fabricated and tested the device in a similar fashion. As seen in Figure 4f, instantaneous discharging is observed when an external load ranging from 1 to 10 kΩ is connected, but the remaining voltage eventually decreases to zero, which is consistent with the capacitive behavior. Thirdly, Cu electrodes were replaced with single‐walled carbon nanotube (SWCNT) electrodes, while maintaining everything else unchanged (i.e., CuCl_2_ as the electrolyte in MXene/PVA hydrogel). The device shows a capacitor‐like discharging behavior, and the voltage eventually drops to zero (Figure S10, Supporting Information). It is concluded that a continuous current output can be achieved only when both CuCl_2_ electrolyte and Cu electrodes are used, strongly suggesting the occurrence of a redox reaction on the electrodes.

The principle for continuous current output is proposed and schematically illustrated in Figure 4g. Initially, a voltage difference is generated when a temperature gradient is applied to the module, due to the thermodiffusion of ions. More anions accumulate on the cold side since it is an N‐type *i*‐TE module. When an external circuit is connected, electrons are driven from the cold side to the hot side through the external circuit, driven by the accumulation of anions on the cold side. The redox reaction in our *i*‐TE module is Cu + Cu^2^⁺ ⇋ Cu^2^⁺ + Cu, a fully reversible process. According to electrochemical theory, the cell potential Δ*V*_cell_ = *E*_red,cathode_ – *E*_red,anode_ (*E*_red,cathode_ and *E*_red,anode_ represent electrode potential of cathode and anode, respectively) is zero in our case due to symmetric reactions on both electrodes, and a small potential can break the symmetry to initiate the reaction. Therefore, the electrons driven to the hot side reduce Cu^2^⁺ to Cu, while Cu on the cold side is oxidized to form Cu^2^⁺. The newly generated Cu^2^⁺ migrates to the hot side due to the concentration gradient. Thus, a complete loop is formed, with electrons flowing through the external circuit and ions moving within the internal MXene/PVA hydrogel.

To investigate the working mechanism in depth, the electrochemical analysis is conducted through cyclic voltammetry (CV) tests. Electrochemical behavior is primarily governed by mass transport in the diffusion layer^[^ ^48^ ^]^ and charge transfer on the electrode surface, as shown in Figure S11a (Supporting Information). A clear understanding of these two processes is essential for accurate analysis of the CV data. During the CV test, as the voltage increases, the concentration gradient in the diffusion layer rises due to the consumption of reactive species, enhancing mass transport efficiency and increasing current. However, the thickness of the diffusion layer also increases with the voltage, eventually resulting in a decline of the concentration gradient that lead to a reduced mass transport efficiency at high voltages.^[^ ^49^ ^]^ Such a transition yields a peak on the CV curve that indicates the maximum mass transport. At the same time, a higher scan rate decreases the thickness of diffusion layer and increases the concentration gradient, which enhance the mass transport efficiency and results in a higher current.^[^ ^50^ ^]^

As shown in Figure S11b (Supporting Information), the CV curves for PM2 with symmetric Cu electrodes exhibit resistive behavior, indicating very effective charge transfer between the electrode and the redox species.^[^ ^51^ ^]^ In addition, the results in Figure S11b (Supporting Information) show that the current slightly increases when the scan rate varies from 10 to 20 mV s^−1^, but saturates with a scan rate beyond 20 mV s^−1^. This suggests the electrode surface reaction kinetics is the limiting factor for enhancing current since the high scan rate indicates a high mass transport process.

The absence of redox peaks indicates that the maximum of mass transport is not reached yet within this potential range. To explore the possible mass transport limit, we have extended the potential window to 3V with a scan rate of 100 mV s^−1^ (Figure S11c, Supporting Information). However, no redox peak is observed, indicating a wide working potential window. This could be due to the unique electrolyte‐electrode system, where the Cu^2+^ in the electrolyte is reduced to Cu on the Cu electrode under negative potential. This newly grown Cu serves as additional redox sites, minimizing the mass transport distance. Similar CV behavior has been observed in several other systems that have symmetric electrodes and reversible redox reactions, such as in symmetric Li^[^ ^52^ ^]^ and Zn electrodes.^[^ ^53^ ^]^

To validate this, a three‐electrode system consisting of a Cu working electrode, Pt counter electrode, and Ag/AgCl reference electrode was set up with 0.01 M CuCl_2_ as the electrolyte. The resultant CVs are shown in Figure S12a (Supporting Information). At lower scan rates (40 and 70 mV s^−1^), a broad local peak appears ≈0.25 V, which is attributed to the mass transport limitations inside the diffusion layer, this peak disappears at a scan rate of 100 mV s^−1^. All curves still exhibit resistive‐like behavior with an increasing trend of current with respect to voltage. We further destroy the symmetrical redox reactions by replacing the electrolyte with 0.01 M NaOH and conducting CV tests under the same three‐electrode system. As shown in Figure S12b (Supporting Information), very typical redox peaks are observed, with the corresponding reactions labeled based on literature references.^[^ ^54^, ^55^ ^]^ The above studies indicate that unique resistive behavior is due to the symmetrical design of our system. Since both electrodes and redox reactions are symmetrical, the reversible reaction Cu^2+^ + 2e^−^ ⇋ Cu is present even at equilibrium state. Consequently, a small positive potential could induce the oxidation and generate more Cu^2^⁺, and a small negative potential can result in reduction reaction and recover Cu^2^⁺ back to Cu, realizing continuous operation even under a very small thermal voltage.

To further verify if the *i*‐TE‐induced voltage can drive the redox reaction, an electrolytic cell (inset of Figure 4h) was prepared with Cu electrodes and a 0.01 M CuCl_2_ electrolyte (same concentration as in the *i*‐TE system). It is clearly seen that, even at a small bias of 2 mV, a high polarization current is produced at the beginning due to the fast response of ions to the electrical field. However, the current doesn't approach zero (polarization equilibrium state) as time goes on (Figure 4h). Instead, it maintains a substantial value and even slightly increases with time, which is strong evidence of the redox reactions. In addition, the color change on the copper electrodes further confirms the redox reactions. Similar corrosion was found in the Cu electrodes in our MXene/PVA‐based *i*‐TE devices, with optical images of reacted Cu electrodes and PM hydrogel being shown in Figure 4i,j and SEM images of the electrodes shown in Figure 4k, confirming the occurrence of chemical reactions during the continuous operation.

It is then straightforward to ask how such a corrosion/reaction impacts the lifetime of our devices. To clarify, one should note that the electrochemical corrosion discussed here is an indispensable process step for realizing our devices in continuous output (i.e., the electrode itself is a part of the redox pair), which is different from a natural corrosion induced by temperature or moisture under environmental conditions (i.e., the device is not in operation), but both of them are critical for devices’ long‐term performance.

The surface of copper is usually covered by a patina layer which protects copper, suggesting that copper is relatively stable at normal environment,^[^ ^56^ ^]^ but it indeed corrodes under some extreme conditions. Here we verify this natural corrosion status of copper under the same environmental conditions (24 °C and RH 65%) as we prepare/test our TE devices. We attached the PVA/MXene hydrogel to copper foil for 12 h and use SEM to check it morphology. As seen in Figure S13 (Supporting Information), no significant abnormality is observed, which is very different from SEM images shown in Figure 4k of the revised manuscript for TE device after extended continuous output, ruling out any influence from natural corrosion.

As for electrochemical corrosion when the system is under continuously operation, we further analyze the corrosion product on the electrodes by using X‐ray photoelectron spectroscopy (XPS). Figure S14a (Supporting Information) exhibits the XPS spectra of Cu2p of cold‐side electrode. In addition to the sharp peaks at 932.4 eV (Cu 2p3/2) and 952.3eV (Cu 2p1/2), which are indicative of metallic copper (Cu^0^),^[^ ^57^, ^58^ ^]^ two more peaks at 934.6 eV and 954.8 eV, along with satellite peaks at corresponding higher binding energies, are observed and they are characteristics of Cu^2^⁺ species. Their presence indicates that Cu^0^ is oxidized into Cu^2+^ due to the electrochemical reaction. However, for hot‐side electrode as shown in Figure S14b (Supporting Information), only metallic peaks are observed, suggesting the hot‐side electrode still metallic state, indicating the regeneration of Cu° from Cu^2+^. These XPS results are consistent with the principle of our TE devices: reduction from Cu^2+^ to Cu^0^ on the hot‐side electrode, while the oxidation of Cu^0^ to Cu^2+^ on the cold‐side electrode. This indicates that the cold‐side electrode could possibly experience severe corrosion after long time working which limits the lifespan of the system. Using Faraday's law of electrolysis,^[^ ^59^ ^]^ the time of complete consumption of a copper foil with a volume of 0.1cm^3^ is calculated to be 1000 h under ideal conditions in a working current of 500 nA (maximum current in currently designed devices). This represents a good lifespan compared to most batteries. A more effective strategy in mitigating the lifetime problem is to operate the devices in an alternative thermal charging mode. Since the oxidation reaction always happens on the code‐side electrode while the reduction reaction occurs on the hot‐side electrode (in our current symmetrical design). The corroded cold‐side electrode can be regenerated if it is shifted to the hot side. In this way, the lifespan could be largely extended.

Another critical factor about lifetime in our devices is the water retention capacity of the hydrogel. The thermoelectric performance of the device is closely linked to the water content of the hydrogel, as water is essential for determining the diffusivity of ions within the hydrogel. To address this issue, a wax coating is used to seal the hydrogel, reducing water evaporation. However, this is only a temporary solution, as it merely slows the rate of water loss rather than addressing the root cause. Future research could explore the use of alternative solvent systems that are less evaporative, such as ionic liquids or polyelectrolytes. These materials exhibit lower vapor pressures and could maintain the necessary ionic conductivity over extended periods without significant water loss, potentially enabling continuous, long‐term operation of the thermoelectric devices.

### 2.5 Applications of MXene/PVA Hydrogel in Sensing and Energy Harvesting

**Figure 5:**
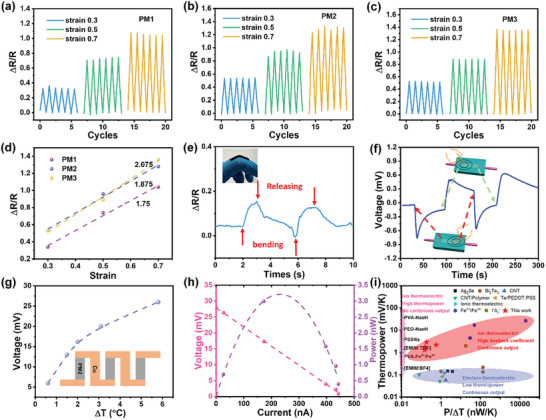
Multifunction of the MXene/PVA hydrogels. The strain sensing (∆*R*/*R*) of a) PM1, b) PM2, and c) PM3 under cyclic stretching strains. d) Gauge factor comparison of PM1, PM2 and PM3. e) Sensing of knuckle bending demonstration. f) Tactile sensing to locate the contact point. g) The output voltage with respect to temperature difference of 3 serially connected PM2 hydrogel‐based devices. h) The output power measurement at a temperature difference of 6 °C. i) A comparison between flexible room temperature *i*‐TE and *e*‐TE devices.

The multifunctionality of MXene/PVA hydrogel has been investigated to demonstrate its potential as a strain sensor, a pinpoint/temperature sensor, and a continuous heat energy harvester. Since 2D MXene sheets are highly conductive but small in size, mechanical deformation will cause the variation of the conductive channels in the hydrogels (for example connection/disconnection of the MXene sheets) and result in a significant change in the hydrogel's resistance. This piezoresistive effect was then utilized as a strain‐sensing mechanism, and ∆*R*/*R* values were used to evaluate the sensitivity. As shown in **Figure** **5**a, ∆*R*/*R* for PM1 reaches 0.33 at a strain of 0.3, and further increases to 0.7 and 1 at strains of 0.5 and 0.7, respectively. PM2 shows even higher ∆*R*/*R* values (0.52, 0.95, and 1.27) at the same strains (Figure 5b), while PM3 exhibits similar values to PM2 but with more stable performance (Figure 5c). The gauge factors are calculated as 1.74 for PM1, 1.875 for PM2, and 2.075 for PM3 (Figure 5d). The increasing trend in the gauge factor suggests that increasing the MXene content enhances the sensitivity of the MXene/PVA hydrogel. An application of the strain sensor is demonstrated for knuckle‐bending sensing. As shown in Figure 5e, finger bending actions produce a unique pattern of *∆R/R* variation with a maximum value of 0.15, both of which could be used to characterize the bending strength and duration.

Besides strain‐sensing via the piezoresistive principle, the MXene/PVA hydrogel can also be used as a tactile sensor to locate the position of a finger or used as a temperature sensor, based on the above‐discussed thermoelectric mechanism. Since the human body is warmer than the environment, the contact of a finger on the MXene/PVA hydrogel will create a small temperature gradient producing a thermoelectrical voltage. As shown in Figure 5f, the voltage increases when a finger contacts one side of the hydrogel and decreases when the finger is removed. Similarly, contacting the other side of the hydrogel causes a voltage with the opposite sign. This means that the sign and magnitude of the voltage/signal depends on the contact location, which offers the spatial resolution to the tactile sensor. Since such a spatial resolution is obtained from a single tactile sensor rather than a sensor array, this mechanism could be used to quickly locate the point of contact of a finger or any warm subject. The same mechanism could be utilized for temperature measurement.

To demonstrate the continuous energy harvesting capability of our MXene/PVA hydrogel, three *i*‐TE devices made of PM2 hydrogel were serially connected, and the generated voltage was measured at various temperature differences with a value of 26 mV being achieved (Figure 5g). The output power at Δ*T* = 6 K was analyzed, revealing a maximum power of 3.2 nW at an output voltage of 14.2 mV and a current of 228 nA (Figure 5h). In the same way, the MXene/PVA hydrogel could be placed on the body to harvest thermal energy from human skin (Figure S15, Supporting Information).

The performance of our MXene/PVA composite is evaluated and compared with other room‐temperature *i*‐TE and *e*‐TE materials (Figure 5i), highlighting its potential for practical applications in thermal energy harvesting. *e*‐TE materials, known for their high electrical conductivity and ability to form closed loops for electron transport, allow for continuous operation and relatively high‐power output per temperature difference. This continuous energy output is advantageous for many electronic applications. However, despite this capability, *e*‐TE materials suffer from a significantly low Seebeck coefficient (thermopower), a key factor in determining thermoelectric efficiency. The low Seebeck coefficient reduces their energy conversion efficiency, making them less suitable for applications that require both high efficiency and adequate voltage output, such as wearable electronics. On the other hand, *i*‐TE materials, which utilize ionic charge carriers instead of electrons, generally exhibit a much higher thermopower. This leads to a higher voltage output, making *i*‐TE devices more practical for applications where higher voltage is essential. However, their inability to provide continuous power output limits their use as a primary energy source. To overcome this limitation, we have developed an innovative device that synergistically combines the thermos‐diffusion‐induced electrolysis effect. This process integrates the Soret effect, which describes mass transport driven by temperature gradients, with the electrolysis effect (consumption of accumulated electrons through redox reaction). By employing this mechanism in a simple, yet efficient, device design, our *i*‐TE device not only achieves a high thermopower but also provides continuous power output. This advancement bridges the performance gap between traditional *i*‐TE and *e*‐TE materials, offering the advantages of both high voltage output and continuous energy supply, making it an ideal candidate for applications like wearable electronics and other low‐power devices.

We manage to include all related research for comparison and categorize them into 4 groups: *e*‐TE, capacitive *i*‐TE, galvanic *i*‐TE, and synergistic *i*‐TE. Overall, *e*‐TE could continuously work, but have a low thermopower. Conventional capacitive *i*‐TE possess very high thermopower but cannot operate continuously. Galvanic *i*‐TE and synergistic *i*‐TE can bridge the gap between. Within the synergistic *i*‐TE group, our system has no voltage threshold and could continuously output in long time, although the output power is moderate due to the simple design. Moving forward, we hope our research inspires further exploration of alternative mechanisms for continuous i‐TE output.

Besides the strategy we propose, the thermogalvanic effect has been well studied to enhance total thermopower, and continuous output could similarly be realized. The aqueous system of Fe(CN)₆⁴⁻/Fe(CN)₆^3^⁻ typically exhibits a thermopower of ≈2–4 mVK^−1^.^[^ ^29^, ^60^ ^]^ Several strategies, such as enhancing the interactions of diffusive ions with the matrix, solvents, and electrodes, have been proposed to improve thermopower.^[^ ^61^, ^62^, ^63^ ^]^ Additionally, by combining the thermo‐galvanic effect of Fe(CN)₆⁴⁻/Fe(CN)₆^3^⁻ with the thermo‐diffusion effect of KCl, a high thermopower of 17 mVK^−1^ was achieved.^[^ ^34^ ^]^ This thermopower outperforms that of our devices. However, it takes more than 30 min for the cell to fully charge thermally. In comparison, our device's charging and response speed to thermal stimulation is fast enough to function as a multifunctional sensor. Most current research on thermogalvanic cells is limited to Fe^3^⁺/Fe^2^⁺ redox pairs and focuses on improving thermopower by incorporating various salts and inorganic solvents. Few studies focus on redox pairs other than Fe^3^⁺/Fe^2^⁺; for example, I⁻/I₃⁻ has been found to have a tunable thermopower ranging from −1.91 to 0.71 mVK^−1^.^[^ ^64^ ^]^ While reducing the water content or adding more organic solvents has been observed to enhance thermopower by reducing the screening effect of polarons, this also significantly reduces ion response time and diffusivity, limiting their application in wearable electronics, where fast response is essential. In another study,^[^ ^65^ ^]^ Zhang et al. addressed this issue by placing the redox species directly on the electrodes rather than in the electrolyte, enabling continuous output and improving mass transport compared to conventional *i*‐TE cells. However, there are two disadvantages: 1) performance deteriorates if the output voltage exceeds the redox window of −0.25 to 0.25V due to mass transfer limitations, and 2) the redox species will be fully consumed if used continuously in the same thermal gradient direction. In conclusion, we are committed to developing a general method for achieving continuous output in traditional *i*‐TE systems and exploring their multifunctionality to meet the growing annual demand for wearable electronics.

## 3 Conclusion

A flexible and multifunctional MXene/PVA hydrogel is successfully developed, and its applications as a continuous *i*‐TE generator and a strain/temperature sensor have been demonstrated. Adding MXene sheets as a binder into PVA hydrogel can hinder the movement of PVA chains, thereby reducing the plastic deformation and increasing the Young's modulus. The MXene/PVA hydrogel infused with CuCl_2_ salt can generate a thermoelectric voltage under a temperature difference via a thermophoresis effect, and a high thermopower of −3.13 mVK^−1^ was achieved. This high value is attributed to the reduced diffusivity of ions through the addition of MXene. The continuous output is realized by combining the thermophoresis effect and the redox reaction of Cu/Cu^2+^ at electrodes via a simple device design, and a three‐serially‐connected TE module can continuously output a maximum power of 3.2 nW with an output voltage of 14.2 mV and a current of 228 nA. Compared to other conventional room‐temperature *i*‐TE systems and *e*‐TE devices, the current MXene/PVA based *i*‐TE system demonstrates a continuous operation with a relatively high voltage, which is more practical in harvesting body heat to power wearable electronics. In addition, the MXene/PVA hydrogel developed here can be utilized to fabricate strain sensors to monitor body motion and tactile/temperature sensors to locate contact positions, further strengthening its potential for use in multifunctional self‐powered sensing.

## 4 Experimental Section

### Synthesis of MXene Dispersion

MXene (Ti_3_C_2_T_x_) was obtained by selectively etching Al layers from the MAX phase (<40 µm, Carbon‐Ukraine Ltd). In a typical process, lithium fluoride (LiF) powder (1.6 g) was dissolved in 9 M HCl solutions (20 mL) and stirred for 10 min. Then, MAX (1 g) was slowly added into this solution to ensure the dispersion of the MAX solids. The dispersion was left to react at 35 °C on a hot plate for 24 h. Afterward, the dispersion was centrifuged (50 mL centrifuging tube) and washed ≈6 times with deionized (DI) water at 3500 rpm until the pH value reached 6. Finally, the sediment was redispersed in DI water and centrifuged for 10 min at 3500 rpm with supernatant collected for further use (concentration is ≈4.4 mg mL^−1^).

### Preparation of MXene/PVA Hydrogel

The Polyvinyl alcohol (PVA, molecular weight, ≈125000) was dissolved in DI water and heated at 90 °C in a water bath for 2 h to prepare a 15% PVA solution. Thereafter, PVA solution (10 mL) was mixed with different amounts of MXene dispersions (100 µL, 300 µL, and 600 µL) after defoaming. The mixture named PM1, PM2, and PM3 was stirred and left in vacuum for defoaming again for 1 h. Then, the mixture was poured into a rectangular cuboid mold and frozen at −25 °C for 12 h to form physical crosslinks between PVA chains. Finally, the frozen hydrogel was thawed at room temperature for 3 h before making a device.

### Fabrication of Ionic Thermoelectric (i‐TE) Device

Two copper foils were attached to a glass slide with a distance of ≈0.5 cm, serving as electrodes. A piece of MXene/PVA hydrogel was cut into strips with the size of 1.5 cm × 0.3 cm × 0.2 cm. The strips (with & without soaking CuCl_2_ or NaCl) were directly attached to the glass slide with both ends connected to the copper electrodes and the contact area on each electrode would be 0.5 cm × 0.3 cm. The entire device was then immersed in molten wax for 2 s and then dried in air to coat the device with a layer of wax in preventing water evaporation during testing. The devices with SWCNT electrodes were also fabricated for testing the effect of different electrodes, and SWCNT film was prepared through vacuum filtration of SWCNT dispersions. To prepare a continuous output *i*‐TE module, three MXene/PVA hydrogel strips were connected in series with copper foils, and the remaining procedures were the same.

### Materials Characterization

MXene films were prepared from synthesized MXene dispersion via vacuum filtration for X‐ray diffraction (XRD) and Raman shift characterization. XRD was conducted using a Bruker D2 Phaser with a Cu kα line (1.54184 Å), and the Raman shift spectrum was measured on a Witec Alpha 300 RAS. The morphology of MXene flakes was observed using a scanning electron microscope (SEM, JEOL JSM‐7610F) and a transmission electron microscope (TEM, Tecnai TEM 200kV). Atomic force microscopy (AFM) images were obtained using an MFP‐3D Origin to investigate the size distribution of MXene flakes. Fourier Transform Infrared Spectroscopy (FTIR) was performed on a Bruker Vertex 80v and element mapping was conducted using a JEOL JSM‐7610F to characterize the MXene/PVA hydrogel. XPS was conducted in Escalate Xi+. Cyclic mechanical stretching and compression of hydrogels are conducted on Instron Universal Testing System.

### Thermoelectric Properties Characterization

To measure the thermopower of the *i*‐TE devices, one end of the module was attached to a temperature‐controlled hot plate and the other end was suspended in air. The voltage difference between the two ends was recorded using a Metrohm Autolab (open circuit voltage measurement module), and the temperatures on the hot and cold sides were measured with a thermocouple. The temperature of the hot plate was room temperature initially and it was set to different values with the time‐dependent voltage difference of the *i*‐TE module recorded. The temperature difference was recorded when voltage reached a steady state. During the transient analysis test, an *i*‐TE device was attached to the hot plate until the voltage stabilized, and then removed from the hot plate for cooling in air. The voltage was recorded throughout the process to monitor the thermal charging and relaxation. For the output power tests, the *i*‐TE module was serially connected to a circuit board with an external resistor. The output power was measured once the voltage reached stable steady under temperature difference. The voltage/current was recorded while varying the resistance of resistor from 330 Ω to 1 MΩ and output power could be calculated as voltage times current. The electrochemical cyclic voltammetry (CV) test was conducted using Metrohm Autolab with two‐electrode method. Counter and reference electrode were connected to one side of the module and working electrode was connected to the other side. A linear voltage scan from −0.6 to 0.6V with a scan rate from 10 to 100 mV s^−1^ was conducted. The electrochemical impedance spectroscopy (EIS) test was conducted using a standardized module in the Autolab software. The voltage amplitude applied was 0.01 V while the frequency varied from 100 kHz to 10 mHz.

### Derivation of Voltage Difference with Respect to Time from Fick Diffusion

The Fick's law of diffusion is governed by a second order differential equation expressed as:

(1) $$\frac{\partial c}{\partial t}=D\frac{\partial^{2}c}{\partial x^{2}}\;$$

where *c* is the concentration, *t* is time, *D* is diffusivity and *x* is the position.

The general solution of 1D diffusion problem from Fourier expansion method for this second order differential equation is:

(2) $$c\left(x,t\right)=\sum_{n\;=\;1}^{\infty }A_{n}\sin\left(n\pi x/L\right)e^{\frac{-n^{2}\pi^{2}Dt}{L^{2}}}\;$$

where *A_n_* is the constant derived from the initial condition, and *L* is the size of the domain.

Therefore, the concentration of ions at hot side is:

(3) $$c\left(x_{hot},t\right)=\sum_{n\;=\;1}^{\infty }A_{n}\sin\left(n\pi x_{hot}/L\right)e^{\frac{-n^{2}\pi^{2}Dt}{L^{2}}}\;$$

And the concentration of ions on the cold side is:

(4) $$c\left(x_{cold},t\right)=\sum_{n\;=\;1}^{\infty }A_{n}\sin\left(n\pi x_{cold}/L\right)e^{\frac{-n^{2}\pi^{2}Dt}{L^{2}}}\;$$

If we consider the major ions that dominate the *i*‐TE effect (Cl^−^), the voltage difference with respect to time could be expressed as:

(5) $$V\left(t\right)=\left(c\left(x_{hot},t\right)-c\left(x_{cold},t\right)\right)\times \mathrm{Volume}\;$$

(6) $$V\left(t\right)=Volume\sum_{n\;=\;1}^{\infty }A_{n}\left(\sin\left(n\pi x_{hot}/L\right)-\sin\left(n\pi x_{cold}/L\right)\right)e^{\frac{-n^{2}\pi^{2}Dt}{L^{2}}}\;$$

Therefore, if we use an exponential function *Y = A + Y_0_e^−X/τ^* to fit this curve, it could be derived that 1/*τ* ∼ *D* which means that *τ* constant could be used to imply the diffusivity of ions.

## Conflict of Interest

The authors declare no conflict of interest.

## Supporting information



Supporting Information

## Data Availability

The data that support the findings of this study are available from the corresponding author upon reasonable request.
