# Supplementary material for: A Multifunctional MXene/PVA Hydrogel as a Continuous Ionic Thermoelectric Generator and a Strain/Temperature Sensor
Source: Small. 2024 Nov 20;21(3):2407529. doi: 10.1002/smll.202407529 (PMC11753485; doi:10.1002/smll.202407529)
Supplement: Supplementary file 1 — Supporting Information [file SMLL-21-2407529-s001.docx]

**Supporting information**

**A Multifunctional MXene/PVA Hydrogel as A Continuous Ionic Thermoelectric Generator and A Strain/Temperature Sensor**

*Dezhuang Ji, Baosong Li, Dawei Zhang, Balamurugan Thirumal Raj, Moh’d Rezeq, Wesley Cantwell, Lianxi Zheng**

Dezhuang Ji, Dawei Zhang, Balamurugan Thirumal Raj, Lianxi Zheng

Department of Mechanical and Nuclear Engineering, Khalifa University of Science and Technology, P.O. Box 127788, Abu Dhabi, United Arab Emirates

Baosong Li, Wesley Cantwell

Department of Aerospace Engineering, Khalifa University of Science and Technology, P.O. Box 127788, Abu Dhabi, United Arab Emirates

Baosong Li, Balamurugan Thirumal Raj, Lianxi Zheng

Research & Innovation Center for Graphene and 2D Materials (RIC-2D), Khalifa University of Science and Technology, P.O. Box 127788, Abu Dhabi, United Arab Emirates

Moh’d Rezeq

Department of Physics, Khalifa University of Science and Technology, P.O. Box 127788, Abu Dhabi, United Arab Emirates; System on Chip Center, Khalifa University of Science and Technology, P.O. Box 127788, Abu Dhabi, United Arab Emirates

Lianxi Zheng

Research and Innovation on CO_2_ and H_2_ Center (RICH), Khalifa University of Science and Technology, P.O. Box 127788, Abu Dhabi, United Arab Emirates

***Corresponding author:** lianxi.zheng@ku.ac.ae (Lianxi Zheng)


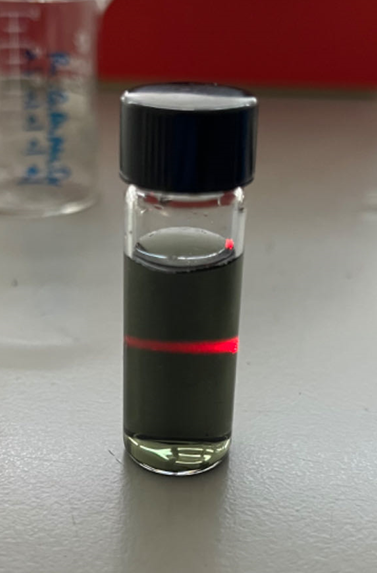


Figure S1: Synthesized MXene dispersion.

Figure S2: Element mapping of MXene/PVA hydrogel.


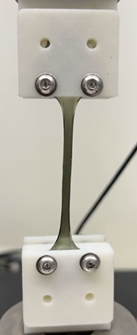


Figure S3: Stretching of the MXene/PVA hydrogel.


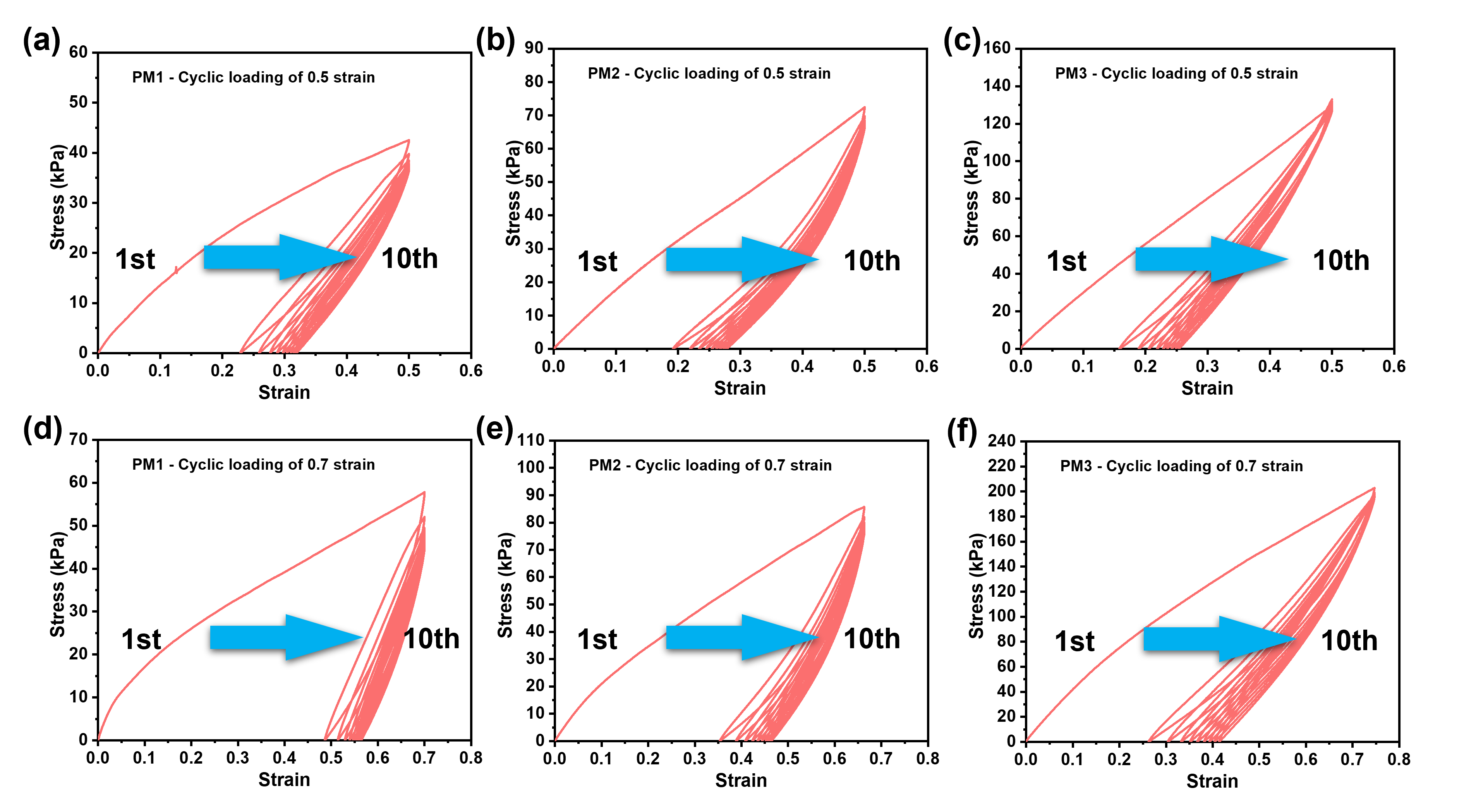


Figure S4: Cyclic stretching at a constant strain of 0.5 for (a) PM1, (b) PM2 and (c) PM3. Cyclic stretching at a constant strain of 0.7 for (d) PM1, (e) PM2 and (f) PM3.


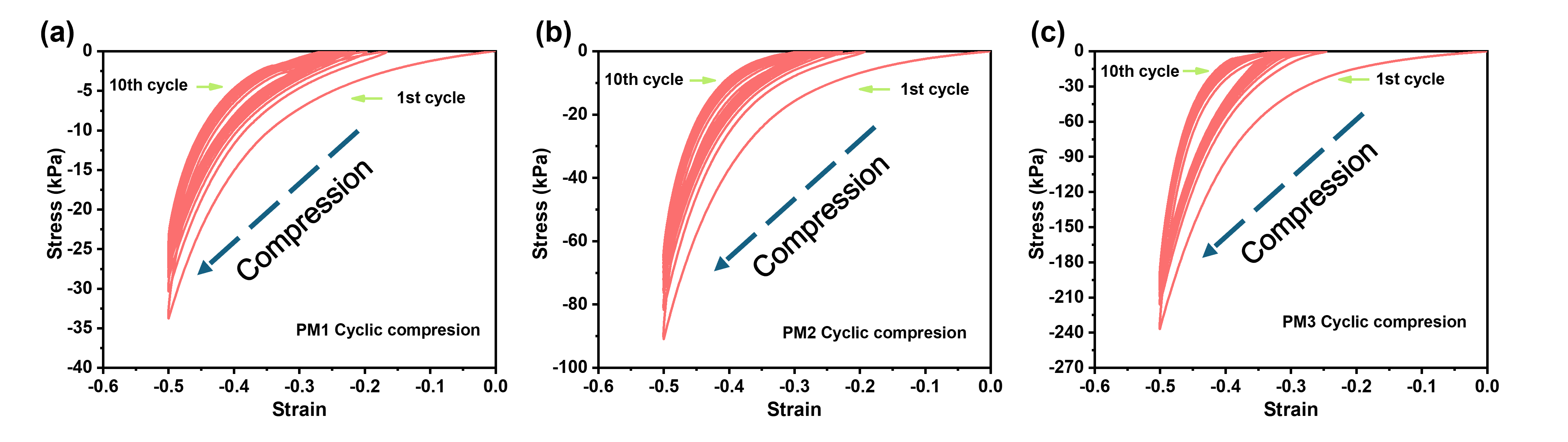


Figure S5: Cyclic compression at a constant strain of 0.5 for (a) PM1, (b) PM2 and (c) PM3.


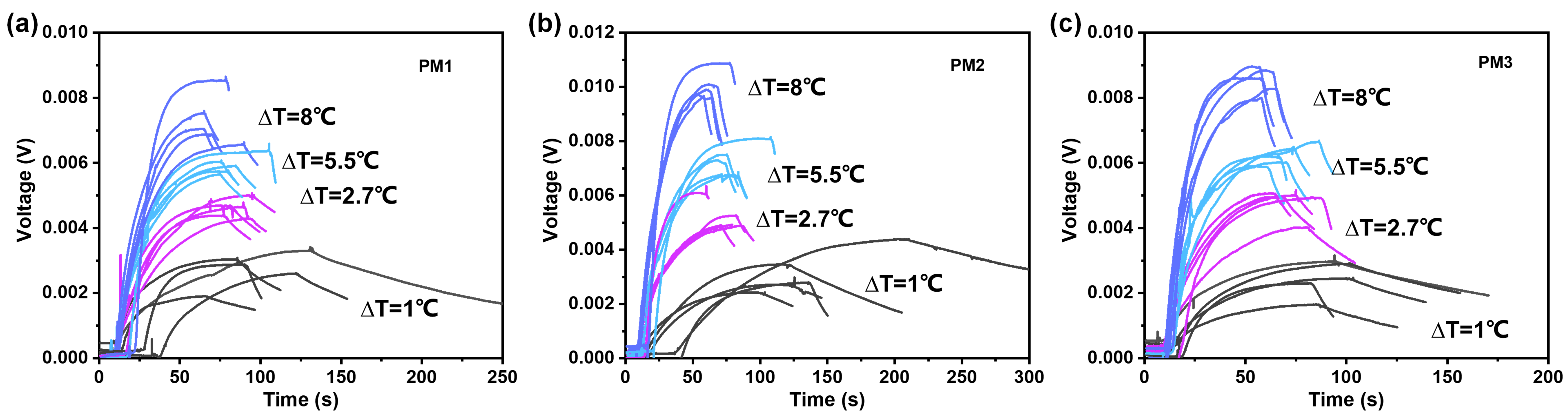


Figure S6: The voltage with respect to time at the temperature difference of 1 °C, 2.7 °C, 5.5 °C and 8 °C of (a) PM1, (b) PM2 and (c) PM3, respectively.


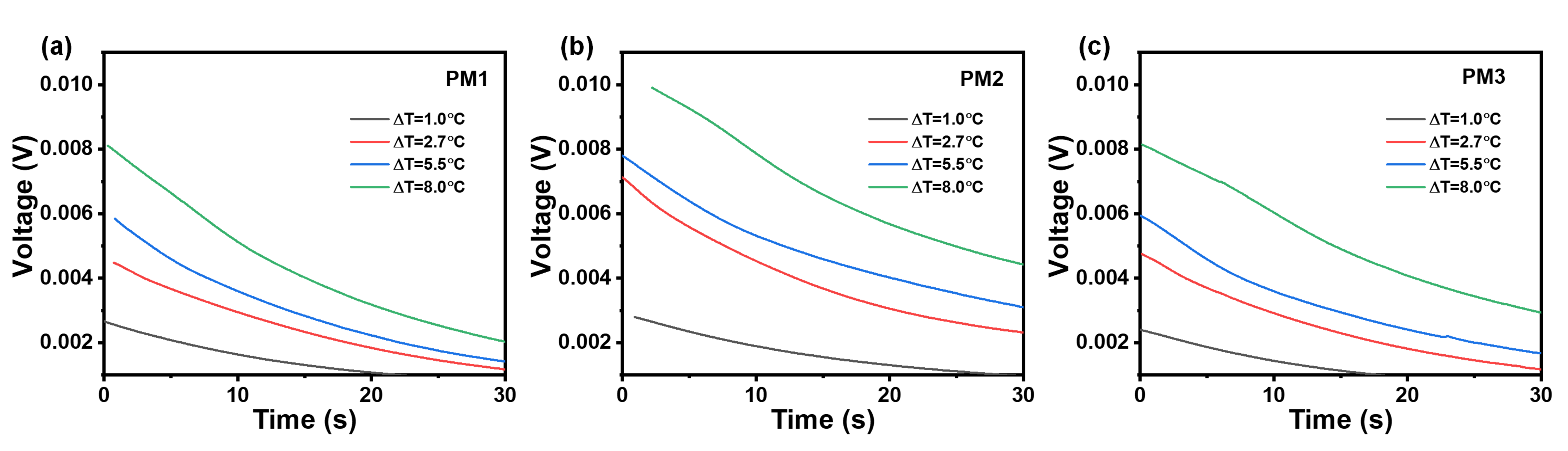


Figure S7: The voltage with respect to time in relaxation process of (a) PM1, (b) PM2 and (c) PM3 from the temperature difference of 1 °C, 2.7 °C, 5.5 °C and 8 °C.

Table S1: τ factors derived from curve fitting equilibrating from different temperatures.

|  | 30$℃$ | 33$℃$ | 37$℃$ | 41$℃$ |
| --- | --- | --- | --- | --- |
| PM1 | 16.5 | 17.2 | 17.8 | 18.8 |
| PM2 | 23.5 | 16.8 | 23.6 | 22.5 |
| PM3 | 16.8 | 19.7 | 17.2 | 24.5 |


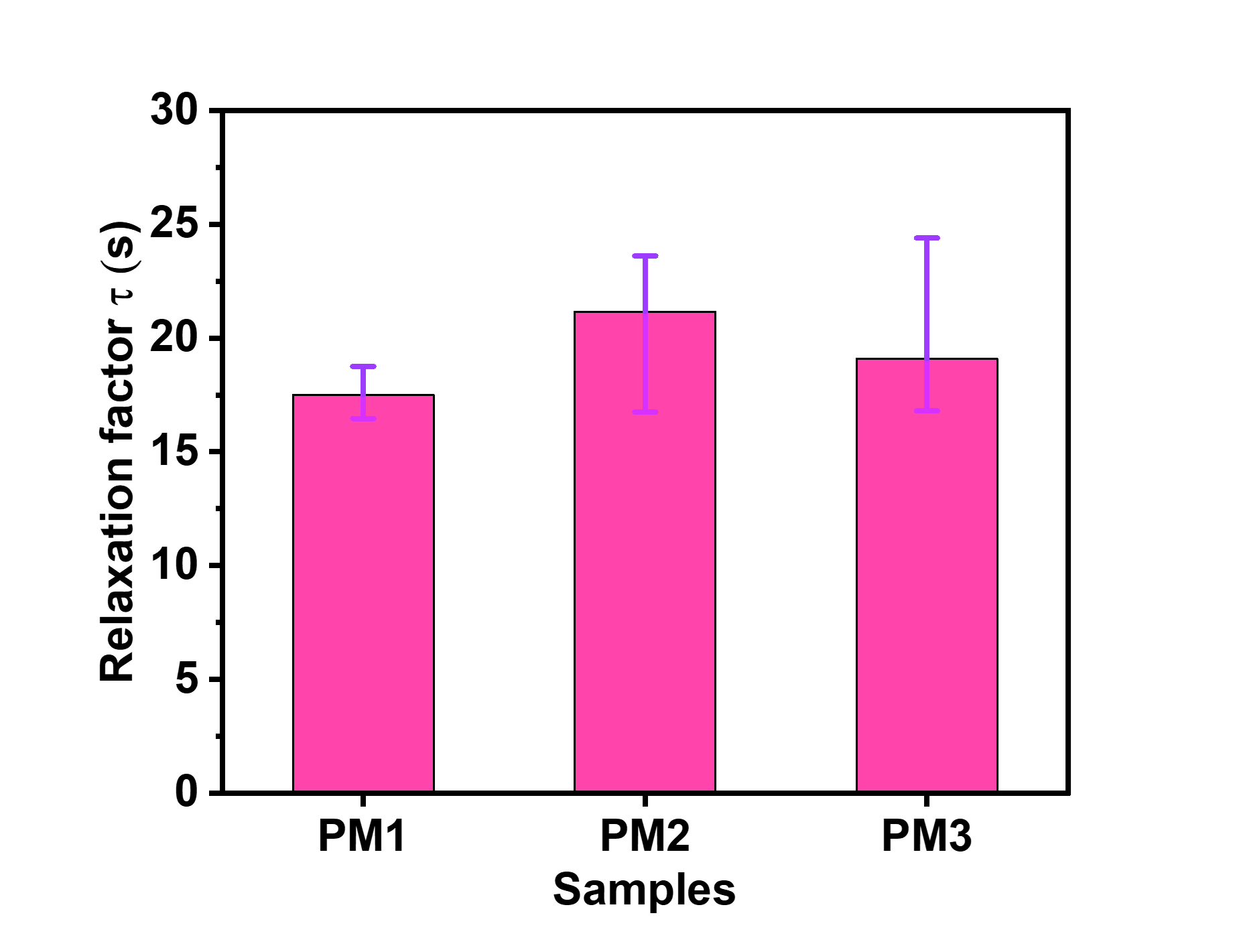


Figure S8: Average relaxation time for PM1, PM2 and PM3.


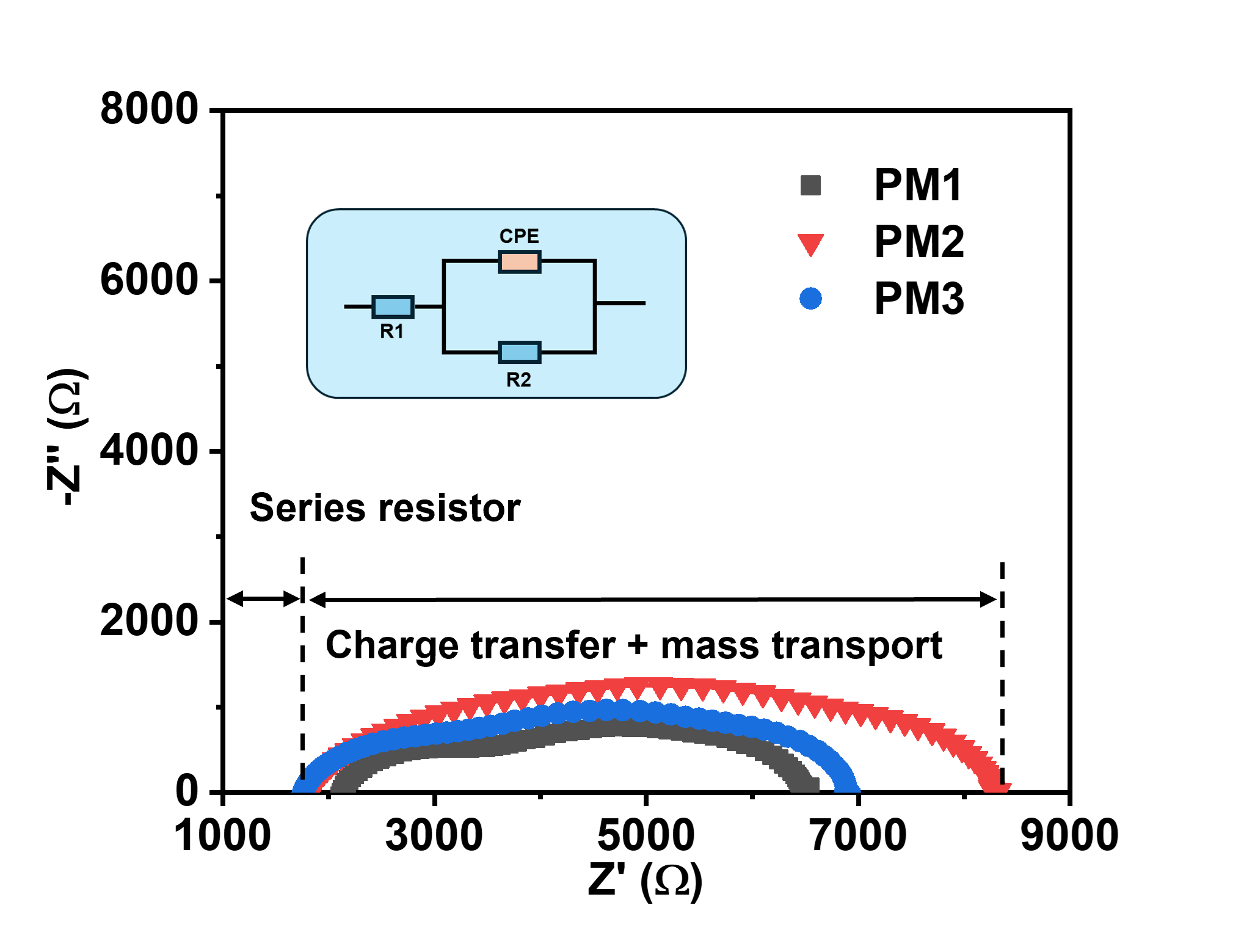


Figure S9: Electrochemical impedance spectroscopy (EIS) of PM1, PM2 and PM3 with equivalent circuit fitting.

Figure S10: Comparison of discharging behavior of PM2 hydrogel with Cu and SWCNT electrode.


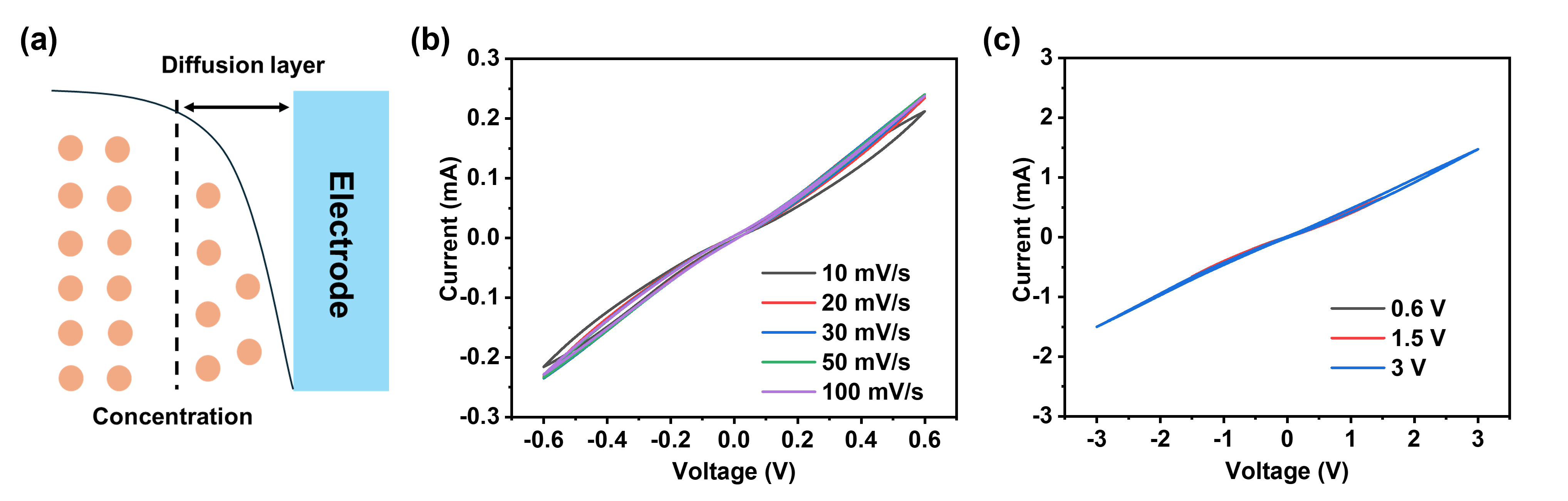


Figure S11: (a) Schematic illustration of diffusion layer on the interface between electrode and electrolyte. (b) The cyclic voltammetry (CV) of PM2 at different scan rates. (c) The CV of PM2 of extended voltage range with the scan rate of 100 mV/s.


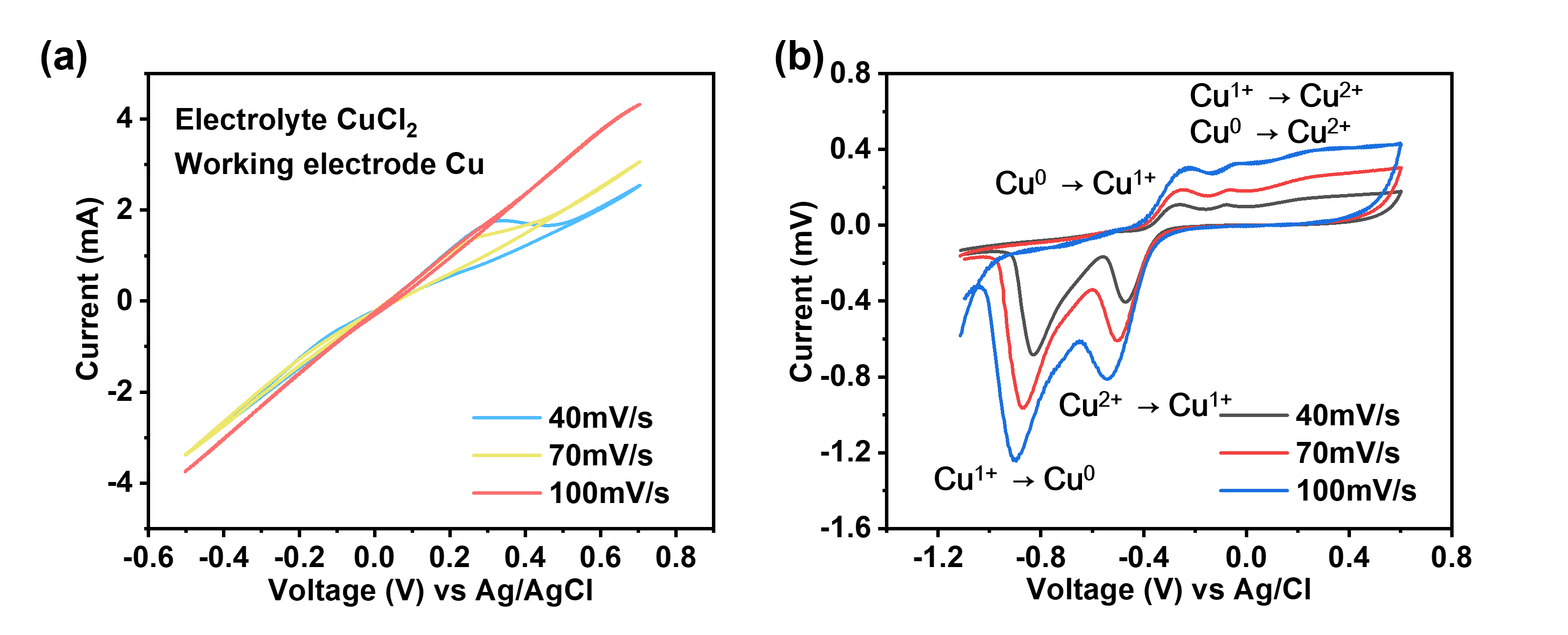


Figure S12: (a) The three-electrode CV of Cu working electrode in the electrolyte of 0.01 M CuCl_2_. (b) The three-electrode CV of Cu working electrode in the electrolyte of 0.01 M NaOH. The counter electrode is Pt and reference electrode is Ag/AgCl in all tests.

Figure S13: SEM image of the surface of copper foil after 12 hours contact with MXene/PVA hydrogel.


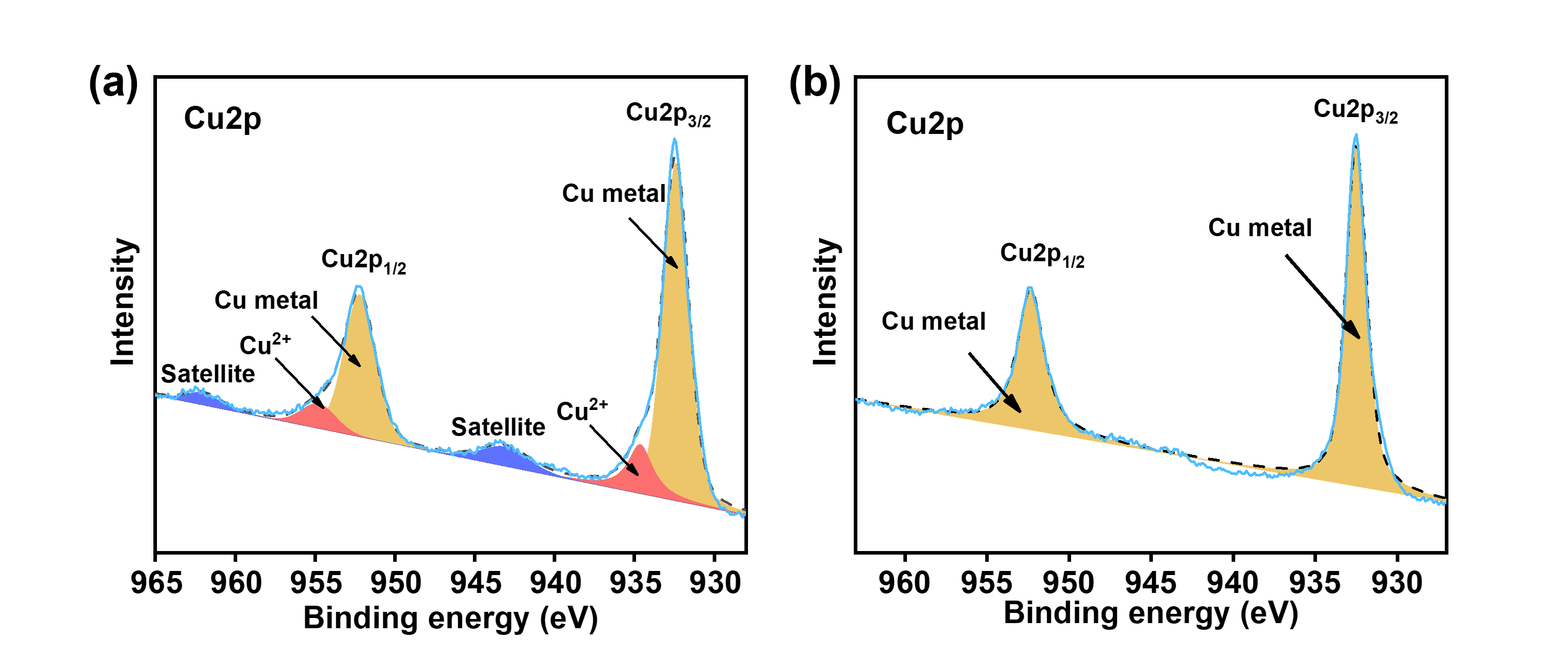


Figure S14: XPS spectra of Cu2p of copper electrode on the (a) cold side and (b) hot side after continuous working.

Figure S15: Demonstration of harvesting of human body heat.
